# Safety and efficacy of canaloplasty versus trabeculectomy in treatment of glaucoma

**DOI:** 10.18632/oncotarget.14757

**Published:** 2017-01-19

**Authors:** Haifeng Liu, Haitao Zhang, Yanhua Li, Han Yu

**Affiliations:** ^1^ Department of Ophthalmology, The Third Affiliated Hospital of Xinxiang Medical University, Xinxiang, Henan, China; ^2^ Department of Ophthalmology, The First Affiliated Hospital of Xinxiang Medical University, Xinxiang, Henan, China

**Keywords:** canaloplasty, trabeculectomy, glaucoma, meta-analysis

## Abstract

We assess the efficacy and safety of canaloplasty and trabeculectomy for treatment of glaucoma. We searched the China National Knowledge Infrastructure, PubMed, Web of Science, and WanFang databases for potentially eligible studies. Pooled risk ratio (RR) with 95% confidence interval (CI) was calculated using random- or fixed-effect models if appropriate. Eight studies were included for meta-analysis. There was no difference in intraocular pressure at 6 months (WMD = 0.97, 95%CI: -0.48-2.41). Intraocular pressure in canaloplasty group 12 months after operation was higher than in trabeculectomy group (WMD = 1.90, 95%CI: 0.12-3.69), *P* < 0.05). The canaloplasty group showed higher success rate than trabeculectomy group (RR = 0.86, 95%CI: 0.77-0.97). The canaloplasty group was more likely to have hyphema (RR = 2.96, 95%CI: 1.51-5.83), *P* < 0.05) than trabeculectomy group (RR = 0.24, 95, CI(0.06-0.89), *P* < 0.05]. The incidence of and hypotony and postoperative choroid abnormalities in canaloplasty group was significantly lower than that in trabeculectomy group (RR = 0.30, 95%CI: 0.11-0.83; RR = 0.24, 95%CI: 0.09-0.66), *P* < 0.05). Both trabeculectomy and canaloplasty can significantly reduce the intraocular pressure in glaucoma patients at 12 months after operation, trabeculectomy leads a more marked IOP decrease than canaloplasty at the cost of a higher complication rate and more demanding for postoperative care.

## INTRODUCTION

Trabeculectomy has long been considered to be the gold standard procedure of lowering intraocular pressure in patients with glaucoma [1, 2]. It involves draining aqueous humor from the anterior chamber into the subconjunctival spaces through a sclerostomy and requires full-thickness penetration of the anterior chamber under a partial-thickness scleral flap [3]. However, trabeculectomy can still be accompanied by some complications such as hyphemia [4–7], hypotony [8, 9], choroidal detachment [10–12], bleb leaks [13–15], and hemorrhaging. These complications are usually increased by using antifibrotics such as 5-fluorouracil, but the rate of short-term succus is relatively low without them [16]. Great efforts have been made for improving the safety and efficiency of glaucoma surgery. Some non-penetrating procedures such as canaloplasty have been reported. In this procedure, intraocular pressure reduction is achieved by opening previously non-functional areas of the outflow system: a 10-0 prolene suture is placed and pulled within Schlemm's canal, thus facilitating outflow through collector channels and aqueous veins [17, 18].

Recently, some researchers conducted some controlled trials to compare the safety and efficacy of canaloplasty versus trabeculectomy in treatment of glaucoma. However, the results still remain to be inconsistent. To our knowledge, the quality and consistency of epidemiological evidence on the topic have not been systematically investigated, which is an important gap in our understanding of safety and efficacy of canaloplasty versus trabeculectomy in treatment of glaucoma. With recently accumulated evidences, therefore, we performed a meta-analysis to compare the safety and efficacy of canaloplasty versus trabeculectomy in treatment of glaucoma. We hope this results could provide some support for clinical practice.

## RESULTS

### Search results and study characteristics

**Figure 1:**
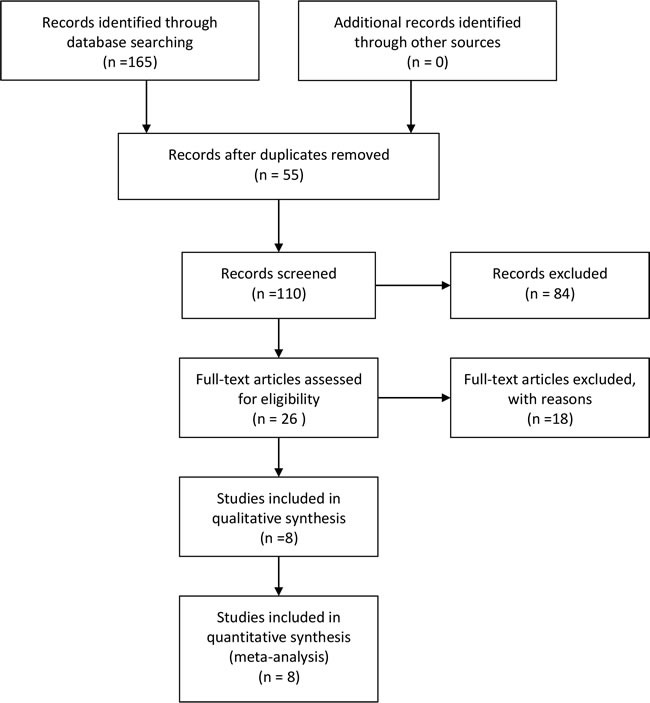
The process of study selection

110 records were left after duplicates removed identified through the initial literature search. After review of the titles and abstracts, we excluded 84 records because they were not related topics. After review of the remaining 28 articles in full, 8 articles met all of the inclusion criteria and entered the final qualitative and quantitative synthesis [19–26]. The selection flow was shown in Figure 1.

The main characteristics of the included studies were presented in Table 1. These studies were published between 2010 to 2015. The sample size was from 30 to 79. 3 studies were from German, 3 ones were from USA, 1 in Italy, and 1 in China. Of 8 studies, 4 are retrospective study and 4 are prospective studies. Only one study was conducted among infant [26], and others were among adults.

**Table 1:** General characteristics of studies included in the meta-analysis

Author	Year	Country	Study design	Age (CP/TE)	Follow-up (month)	Sample size (CP/TE)	NOS
Schoenberg	2015	USA	Prospective cohort	65.7/67.1	12	36/41	7
Quaranta	2014	Italy	Prospective cohort	63.8/63.0	12	26/26	6
Thederan	2014	German	Retrospective cohort	65.6/68.6	12	18/22	7
Bruggemann	2013	German	Retrospective cohort	58.7/59.5	12	15/15	7
Matlach	2013	German	Prospective cohort	63.2/60.1	12	19/20	8
Bruggemann	2012	USA	Retrospective cohort	-/-	12	21/48	6
Ayyala	2011	USA	Prospective cohort	65.6/68.6	12	33/46	8
Huang	2010	China	Prospective cohort	25.2/23.5	12	23/28	7

* CP=Canaloplasty, TE=Trabeculectomy, NOS=Newcastle-Ottawa Scale

### Assessment of quality

The NOS of all included studies ranged from 6 to 8 points. The average NOS score of the studies was 7. An additional file shows this in more detail (Supplementary Table S1).

### Pooled results

#### Intraocular pressure

**Figure 2:**
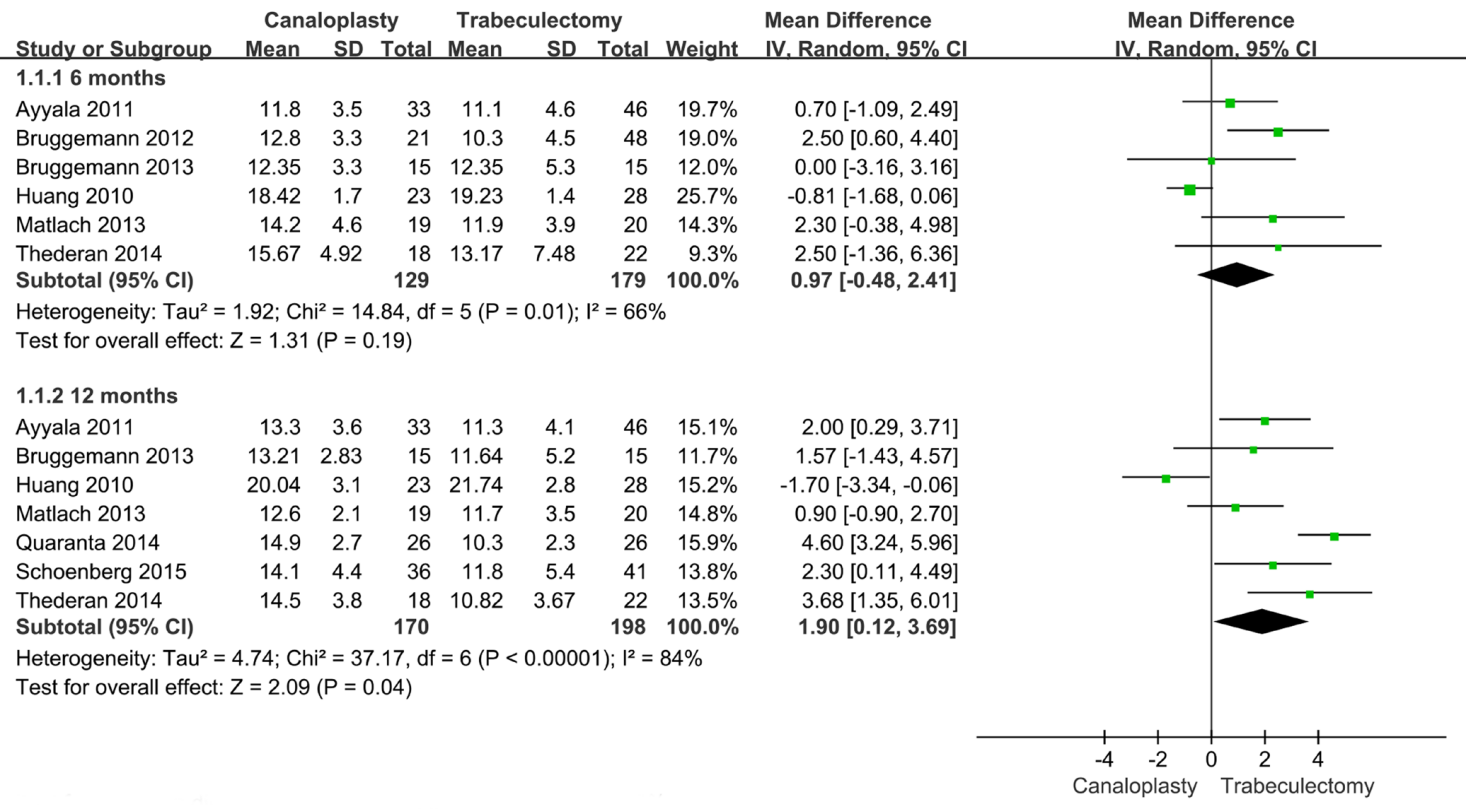
Forest plot of intraocular pressure at 6 and 12 months after operations

Figure 2 presents the pooled intraocular pressure results. This process includes 129 patients with canaloplasty and 179 patients with trabeculectomy. The random-effects models were used at 6 and 12 months after operation because the heterogeneity were high (*I*2 = 66% and *I*2 = 84%). At 6 months, intraocular pressure of canaloplasty group was not prior to that in trabeculectomy group (WMD = 0.97, 95%CI: -0.48-2.41, Figure 1). The differences showed no significant. However, intraocular pressure of canaloplasty group were higher than in trabeculectomy group (WMD = 1.90, 95%CI: 0.12-3.69, Figure 2).

#### Success rate

**Figure 3:**
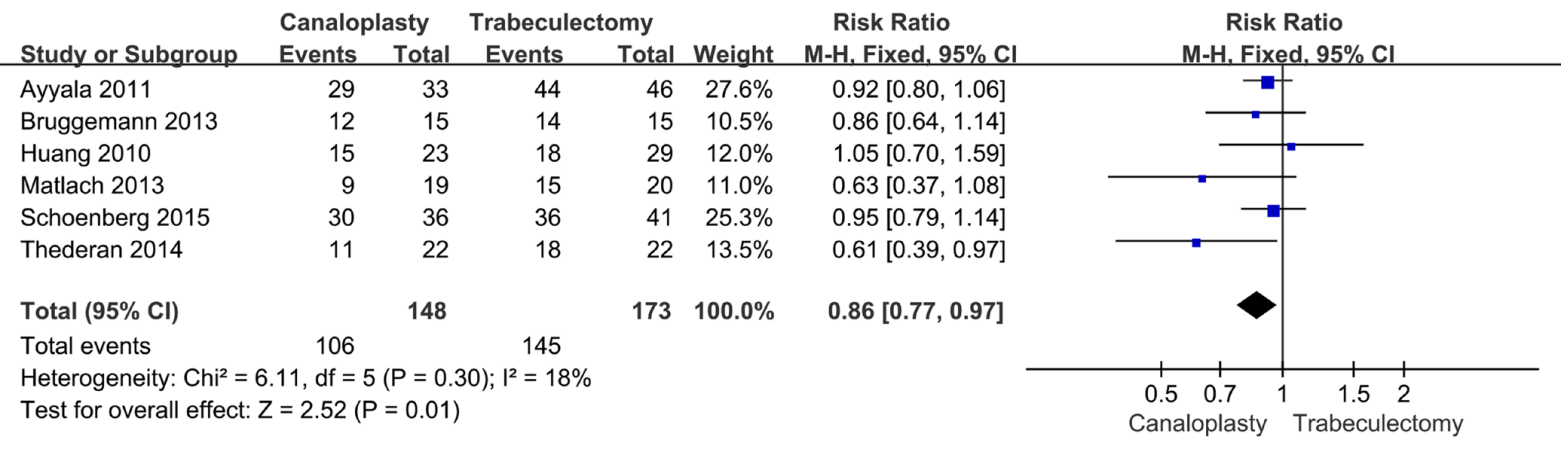
Succus rate of intraocular pressure control between two group

Six studies reported the succus rate of intraocular pressure control, including 148 canaloplasty patients and 173 trabeculectomy patients. The results from fixed-effect models showed that success rate of canaloplasty group was lower than that of trabeculectomy group (RR = 0.86, 95%CI: 0.77-0.97, *P* = 0.010, Figure 3), with low heterogeneity (*I*2 = 18%).

#### Complications

**Figure 4:**
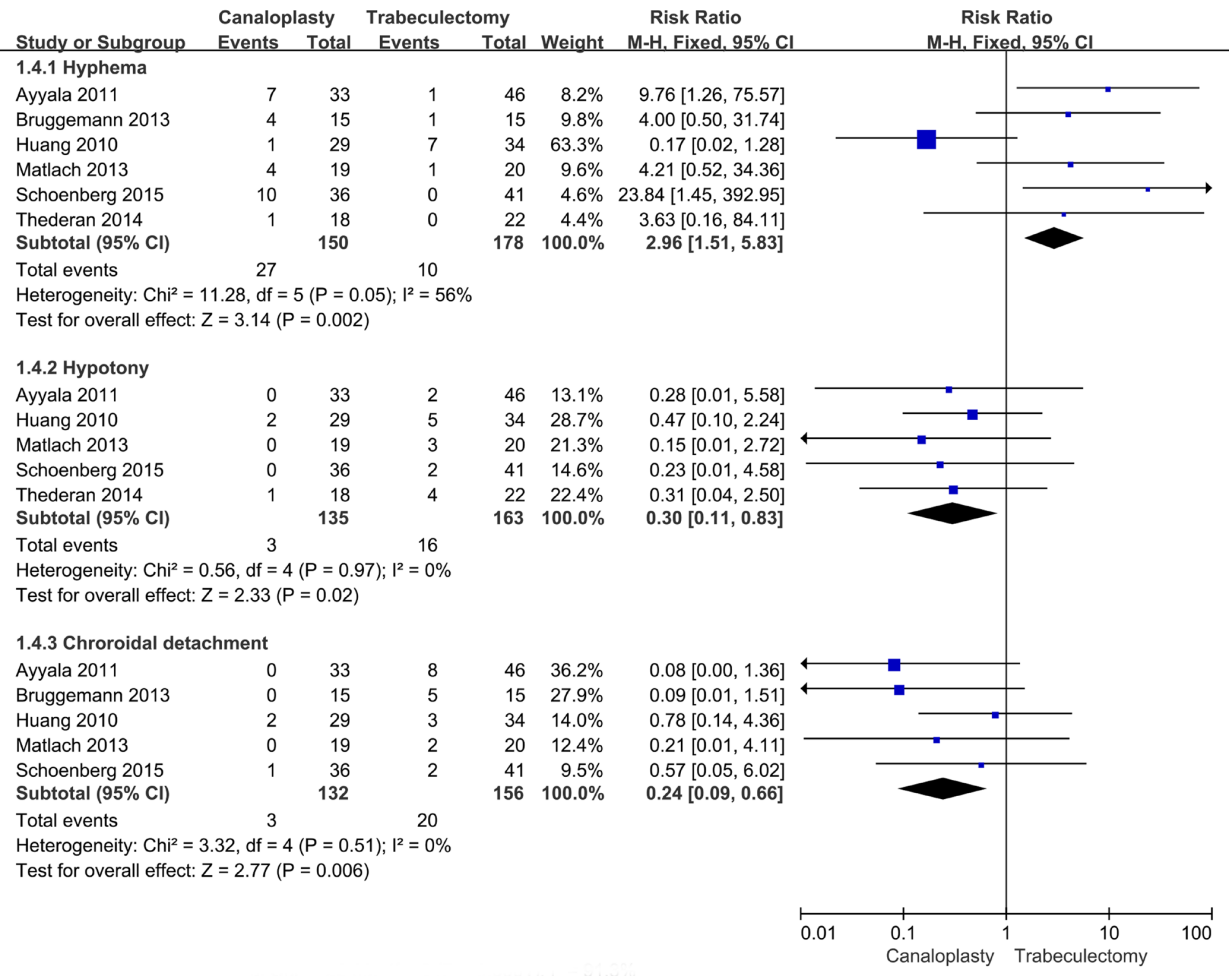
Forest plot of complications risk between two group

Six articles reported that adverse reactions and complications during follow-up, including hyphema, hypotony, and choroidal detachment. Five studies reported that incidence rate of hyphema. The heterogeneity within studies is low (*I*2 = 56%, *I*2 = 0.0%, *I*2 = 0.0%), and the fixed-effects models were conducted for these pooled results. Our results show canaloplasty group has a high risk of hyphema compared with trabeculectomy group (RR = 2.96, 95%CI: 1.51-5.83). However, canaloplasty group has lower risk of hypotony and choroidal detachment (RR = 0.30, 95%CI: 0.11-0.83; RR = 0.24, 95%CI: 0.09-0.66) compared with trabeculectomy group. The Figure 4 gives the specific results.

### Sensitivity analysis and publication bias

**Figure 5:**
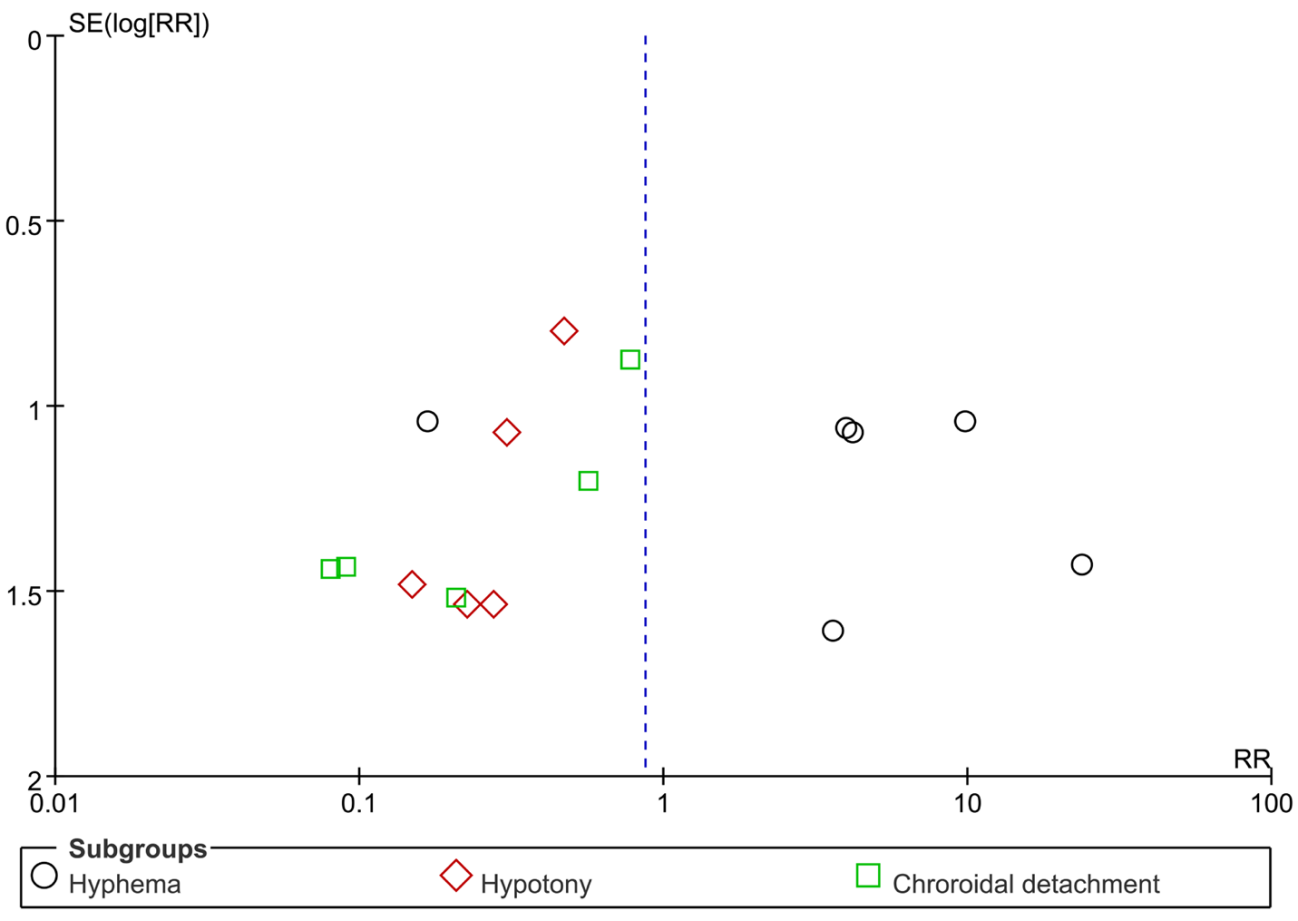
Funnel plot for publication bias assessment

We conducted sensitivity analysis through excluding some certain studies. The results did not alter exactly (Supplementary Figure 1). We use the Egger's and Begger’ s to examine the publication. Egger's test suggested that publication bias did not exist (t = 0.560, *P =* 0.600). Also, the Begger's test give a similar result (Z = 0.300, *P =* 0.764). The results are presented in Figure 5.

## DISCUSSION

The present study found that trabeculectomy could reduce the intraocular pressure more obviously than canaloplasty at 12 months after operation. Trabeculectomy group have higher succus rate of intraocular pressure control while canaloplasty group are more likely to have hyphema compared with trabeculectomy group, but less possible to have hypotony and choroidal detachment.

Reducing intraocular pressure for glaucoma patients is the primary concern of operation therapy, with the aim of alleviating the optic nerve pressure. Reducing intraocular pressure is quite important for glaucoma treatment. Heijl reported that glaucoma progression had a 19 percent reduced risk when intraocular pressure decrease by 1mm Hg. Trabeculectomy operation is the standard treatment methods, but many complications would appear after operation and require more postoperative intervention. In canaloplasty procedure, the increased physiological drainage of trabecular meshwork archived by opening or expanding Schlemm's canal, without functional filtering bleb [27]. This procedure can avoid postoperative infection of filtering bleb and nursing, and theoretically can reduce the occurrence of postoperative complications and intervention therapy. If canaloplasty method can be proved to have similar of reducing intraocular pressure like trabeculectomy, and have less complications, these will have great important clinical significance. Currently, some clinical controlled trials are conducted to evaluate the safety and efficacy of comparing the canaloplasty versus trabeculectomy. However, the results remain inconsistent. Schoenberg reported that canaloplasty and trabeculectomy both achieved significant reduction in intraocular pressure and improvement in VA at 12 months with comparable success rates. trabeculectomy achieved a statistically greater median percentage decrease in intraocular pressure, but the 2 procedures resulted in comparable mean IOP at 12 months [19]. Thederan reported that the mean intraocular pressure in the trabeculectomy and canaloplasty groups reduced from 23.91 to 10.82 mmHg and from 23.68 mmHg to 14.50 mmHg, respectively. The postoperative complications and interventions between the two groups did not reveal a statistically significant difference. Complete success was achieved in 18 eyes (81.8 %) after trabeculectomy and in 11 eyes (50.0 %) after canaloplasty [21]. Ayyala, found that the mean percentage reduction in intraocular pressure from preoperative values at 12 months after surgery was 32% for the canaloplasty group compared with 43% for the trabeculectomy group. The median reduction in the number of medications at 12 months’ follow-up was 3 in the trabeculectomy group and 2 in the canaloplasty group. But the differences were not significant. A higher percentage of patients treated with canaloplasty than trabeculectomy (36% vs. 20%) required postoperative medications, although this also did not attain significance (*P* = 0.12). Failure rate based on intraocular pressure was 12.1% for the canaloplasty group and 4.3% for the trabeculectomy group. There was still no difference in surgical failure rates between the canaloplasty and trabeculectomy [25].

The present study gives more powerful results Our findings provide support for clinical practice. Based on the present evidences, we propose that trabeculectomy operation should be applied for glaucoma patients with relatively high normal intraocular pressure. This method could improve the succus rate. Our results found that patients who received trabeculectomy had a higher risk of hypotony and choroidal detachment, indicating that canaloplasty operation could be used for patients who tended to have such complications like this. Although canaloplasty group have a higher risk of hyphema compared with trabeculectomy group, this complication usually did not carry out intervention because hyphema could be absorbed 1-3 weeks after operation [28].

Strengths of this meta-analysis included its validated and systematic review methods following PRISM guidelines and its high quality of included studies, with an average score of 7 allowing for the impossibility of one single study. These strengths make it possible for the present study to minimize the potential bias as much as possible, which is of great importance in the study design of observational studies. However, there are still several limitations needed to be addressed. First, the study number in this meta-analysis is limited, it is possible to have some bias. Second, six of included studies are from Caucasian, and only 2 of them are from Asian. The present results are limited in applying other population setting. Third, the assessment of effective are between 6 and 12 months after operation. More long-term follow-up are required to compare the long-term therapeutic effect. Finally, we can't assess the impact of the other clinically meaningful end points, such as automated perimetry, frequency doubling technology perimetry, scanning laser polarimetry, and confocal scanning laser ophthalmoscopy, because of sparse and inconsistent reporting across studies.

In conclusion, this meta-analysis of eight studies suggests that trabeculectomy method can significantly reduce the intraocular pressure better than canaloplasty method in glaucoma patients after operation, trabeculectomy leads a more marked IOP decrease than canaloplasty at the cost of a higher complication rate and more demanding for postoperative care.

## MATERIALS AND METHODS

We performed this study in accordance with the Preferred Reporting Items for Systematic Reviews and Meta-Analyses (PRISMA) statement. No ethical statements are required for this study (Table S2).

### Literature search

We searched China National Knowledge Infrastructure (CNKI), PubMed, Web of Science, and WanFang databases from inception to December 2016 for eligible studies, using the following Medical Subject Heading (MeSH) terms and keywords: canaloplasty, trabeculectomy, and glaucoma. Language was restricted in English and Chinese. We also checked the reference lists of previous related reviews and included studies for potentially eligible studies.

### Selection criteria

We included the published studies that must meet the following criteria: (1) Type of study design: Clinical study with controls (No matter whether to use blind method or lost follow-up). (2) Study subject: patients with glaucoma. (3) Intervention: study with canaloplasty and trabeculectomy operation. (4) Outcomes: study with rate of efficient, intraocular pressure at 6 and 12 months after operation, higher success rate, and appearance of complications (hyphema, hypotony, and choroidal detachment).

The following studies are excluded: (1) patients with previous glaucoma operation or other intraocular surgery. (2) study cannot provide sufficient data for extraction and analyses or can't obtain original data from authors. (3) replicates articles are excluded.

Two investigators independently conducted the initial search, deleted duplicate records, screened the titles and abstracts for relevance and identified records as included, excluded or requiring further assessment.

### Data extraction and assessment of quality

The following information was extracted from each study: the first author, publication year, country, study design, age (control and trial), period of follow-up, and sample size (two groups), intraocular pressure at 6 and 12 months after operation, success rate of operation, and some complications (hyphema, hypotony, and choroidal detachment) shown in results part.

The studies included in the meta-analysis were prospective or retrospective studies. We used Newcastle-Ottawa Scales (NOS) to assess the study quality [29]. The NOS includes 3 categories (selection, comparability and outcome) with 9 items. We evaluated the quality of studies as the following criteria: priority (7-8 adequate items), high quality (equal or more than 5 items), low quality (less than 5 items) and extreme low quality (no description of study methods).

### Statistical analysis

We use the Review Manager software (RevMan version 5.2; Nordic Cochrane Centre, Cochrane Collaboration) and Stata 12.0 (Stata Corporation, College Station, TX, USA) to complete all analyses. We assessed the heterogeneity within the studies using the *I*2 statistic and Cochran Q Chi-square test [30]. We considered heterogeneity to be substantial if the *I*2 value was greater than 50%. We pooled the data using the random-effects models or fixed-effects models. For continuous variables, we calculated standardized mean differences (WMDs) with 95% CIs. For dichotomous data, we calculated relative risks (RRs) with 95% confidence intervals (CIs). Sensitivity analyses and publication bias were evaluated using the Stata 12.0. A *p* value of less than 0.05 was considered to be statistically significant.

## SUPPLEMENTARY MATERIALS FIGURES AND TABLES



## Abbreviations

CNKI: China National Knowledge Infrastructure
MsSH: Medical Subject Heading
NOS: Newcastle-Ottawa Scales
WMD: weighted mean difference
RR: relative risk
CI: confidence interval
PRISMA: Preferred Reporting Items for Systematic Reviews and Meta-Analyses
